# Recommending blue ocean technologies for subcontractors: A framework based on business reports of prime contractors and patents

**DOI:** 10.1371/journal.pone.0256157

**Published:** 2021-08-18

**Authors:** Hyunwoo Woo, Sun Woo Lim, So Young Sohn

**Affiliations:** 1 Department of Industrial Engineering, Yonsei University, Shinchon-dong, Seoul, Republic of Korea; 2 Department of Applied Statistics, Yonsei University, Shinchon-dong, Seoul, Republic of Korea; Northumbria University, UNITED KINGDOM

## Abstract

Subcontractors depend heavily on their prime contractor and thus find it very risky to enter a new business on their own. This study proposes a framework for these subcontractors to develop blue ocean technologies related to their prime contractor. First, the primary technologies predicted to be promising are extracted from the business reports of the prime contractor. Sub-technologies are then selected through a patent-based search using keywords and International Patent Classification codes of the primary technologies. From them, blue ocean technologies are proposed by optimizing the weighted mean of the min-max normalized market value, degree of competition in the technology market, and subcontractors’ potential technological capabilities for each sub-technology. This study shows that subcontractors can enhance their technology competitiveness by finding a low-risk blue ocean technology. Our empirical research on the subcontractors of a semiconductor firm identified technological patent fields for them to pursue. From our framework, subcontractors can identify blue ocean technologies by considering their prime contractor’s future industrial areas and technologies of interest as well as their own technological capabilities. Furthermore, the prime contractors can gain the synergy effect of technology expansion through cooperation.

## 1. Introduction

Subcontracting, also called outsourcing, is an efficient and economical method for prime contractors to access the resources they need [1,2]. Subcontractors are useful partners for prime contractors to diversify their market risks by reducing their operating costs and increasing their competitive advantage [3]. Subcontractors perform tasks that their prime contractors cannot carry out efficiently by themselves, using their unique technology in a more cost-effective manner [4]. Thus, subcontracting is an important business option for prime contractors to flexibly respond to dynamically changing markets and save money on hiring, training, and managing workers [5–7]. Many prime contractors still focus on their representative technology, but they increasingly depend on subcontractors for additional technology inputs [8,9] and even on high-level subcontractors when new technologies are involved [10]. According to the radical innovation theory, which emphasizes the scalability of knowledge including gaining a new technology as a result of R&D activities [11], prime contractors such as large companies are passive about the radical innovation because they tend to hedge risks due to their hierarchical organizational structure and large scale [12]. However, subcontractors like SMEs have a better chance to try radical innovation than their prime contractors, and the technological development of the subcontractors helps their prime contractors expanding their technologies as well. Therefore, subcontractors have become key factors of technological business and should build up their technical capabilities and prepare for both their own and their prime contractors’ future businesses through technological innovation [13,14].

However, subcontractors cannot readily engage in technology development because the prosperity and growth of their businesses directly depend on the bid opportunities provided by prime contractors [15]. In fact, subcontractors and their prime contractors are asymmetrical in power relations, and prime contractors do not have to pay close attention to the growth of their subcontractors [16,17]. Subcontractors typically take no initiative to learn new technologies and tend to depend permanently on their prime contractors, without deepening their technology base [18]. Thus, they are more likely to adhere to their existent knowledge base rather than attempt to learn something new, thus hindering their innovation [19]. When subcontractors and their prime contractors do not realize that they can contribute to each other’s innovation, much of their talent is wasted [20].

As subcontracting is known as a governance which is efficient for the diffusion of new technology [21], subcontractors need to innovate, gain technological competitiveness, and cooperate with their prime contractors actively in a win-win relationship by investing in technologies and resources. It would be difficult for subcontractors to keep pace with the new technology demands and rapidly changing trends by themselves [22]. On the other hand, the risk can be reduced as subcontractors grow along the direction of their prime contractors. Therefore, we need to consider the innovation capacity of subcontractors as well as the innovation challenges of their prime contractors [23]. However, previous studies on subcontractors’ technology development generally dealt with the relationship between subcontractors and prime contractors, without carefully considering subcontractors’ technological followership in which subcontractors keep pace with new technology demands and its trend in line with the interests of prime contractors. For examples, Cao and Wang [24] showed that a subcontractor’s innovative technology makes more positive relationship between a subcontractor and its prime contractor. Plants which have had experience of subcontracting can adopt a high level of manufacturing technology more easily than those which have not [21].

**Fig 1:**
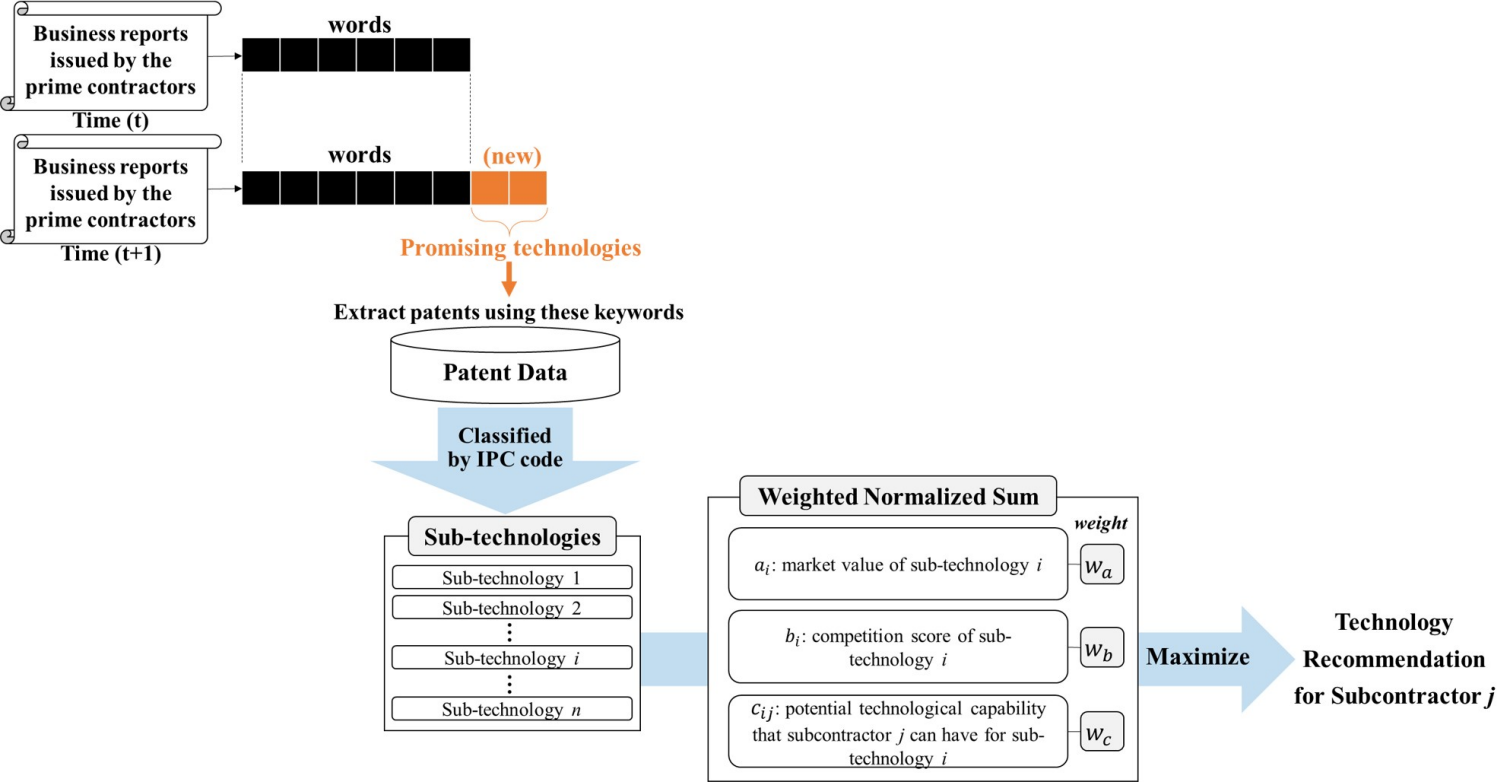
Flow chart of the proposed framework.

In order to provide practical assistance to subcontractors which are preparing for the future, it is necessary to consider their technological followership. This study provides a framework, as shown in Fig 1, for subcontractors to identify advanced technologies by considering not only their technological capabilities and market competition, but also the new technological areas that their prime contractors are seeking to develop. As subcontractors are highly dependent on their prime contractors [13,16,18], it would be risky for them to set the direction of new technologies to be developed on their own. Since our approach satisfies the stakeholders’ innovation needs, it would enable subcontractors to reduce the uncertainty of investment costs and make significant contributions to their supply chain [25]. Our approach first identifies the promising technologies from the business reports of the prime contractor which contains the future plans. Sub-technologies of the promising technologies can be found through a patent-based search using the keywords as well as International Patent Classification (IPC) codes of the promising technologies. In order to attract the prime contractor, the subcontractor needs to be equipped with valuable technologies which do not have many competitors. We call them blue ocean technologies. To select blue ocean technologies among the sub-technologies, we consider three patent-based factors: the sub-technologies’ market value, sub-technologies’ degree of competition in the technology market, and subcontractors’ potential technological capability for each sub-technology which represents how similar the subcontractors’ technologies are to their prime contractors’ technologies in terms of their patents. Blue ocean technologies are selected using an integer programming to maximize the weighted sum of the three factors.

This paper is organized as follows. Section 2 presents the related literature. Section 3 describes the framework of the study. Section 4 shows an empirical study applying the proposed framework, and Section 5 discusses our results in relation to the existing studies. Section 6 concludes the paper with a summary.

## 2. Literature review

Companies continue to pursue new trends and develop appropriate skills to survive in the market [26]. In this technology-driven economy, with corporate innovation growing rapidly, companies need to decide on technology adoption continuously [27]. The advanced technologies adopted bring significant benefits to companies along with innovation [28,29] and induce the interest of investors and governments [30]. However, the fluctuating technology market makes it impossible for a company to perform all its tasks using its internal R&D alone [22]. Subcontractors can solve this problem by working as partners to their prime contractors in the technology market. Subcontracting is a commercial contract wherein a subcontractor carries out the prime contractor’s responsibilities and duties [31]. This has been in use for a long time, with wider implementation in recent days [32]. The advantage of subcontracting is that subcontractors are specialized in a few tasks, and can thus deal with the tasks of their prime contractors more efficiently and rapidly [1]. As the value of subcontracting increases, we need to pay attention to technological development or innovation to subcontractors.

Several studies have examined various aspects of subcontracting because this is an important issue. They mainly focused on the subcontractor’s bidding [33,34], the management relations between subcontractors and prime contractors [15], and investment from the perspective of the prime contractor [35–37]. Since innovation is important for a continuously successful business, in addition to efficiency, some other factors such as the subcontractors’ technological knowledge or advanced technology have also become significant issues in subcontracting [38]. In fact, the more innovative and fundamental the subcontractors’ technologies are, the less is the subcontractors’ uncertainty and the more positive is the development in their relationship with their prime contractors [24]. Furthermore, subcontractors adopting high technology levels evolve by developing their technological competencies [38]. Sturgeon [39] argued that subcontractors play a more leading technological role than prime contractors. Thus, subcontractors need to invest in technological innovation [40] and improve their technological competitiveness by developing additional resources [41]. However, subcontractors prefer familiar tasks instead and hamper their innovation, unaware of their potential capabilities [19,20]. Furthermore, subcontractors usually tend to rely heavily on their prime contractors [16,18,41] and take no initiative on their own technology development. Therefore, subcontractors need to be motivated to develop new technologies, but it is hard to find studies which are concerned with subcontractors’ technological development. Cao and Wang [24] argued that technological innovation of subcontractors has a positive correlation with the relationship between subcontractors and their prime contractors. Pardo and Rama [21] argued that a manufacturing factory with experience in subcontracting adopts a higher level of technology than those which have not. However, even if these studies dealt with the topic of technological development or technological innovation, they did not take into account the specificity of subcontractors’ technological followership to their prime contractors.

As subcontracting is a kind of auction in which a prime contractor selects a small number of subcontractors among many candidates, it can be helpful for subcontractors to use blue ocean strategy (BOS) for winning the subcontracting process and reducing their risk in developing new technologies. BOS is a method to find low-competitive markets and pursue value innovation, differentiation, and low-cost productivity [42]. It shows companies the possibility of more profitable and rapid growth in an innovative and developmental market compared to that in the existing competitive and crowded market [43]. To determine the feasibility and profitability of commercializing new technologies, we need to evaluate them both subjectively and objectively [44]. The former is based on expert knowledge and experience, whereas the latter is based on quantitative evaluation. Therefore, this study recommends that subcontractors choose technologies based on their prime contractor’s business reports as a qualitative method and then analyze those technologies quantitatively using patent data. Patent data comprise a collection of the technical capabilities and expertise in new technology [45]. Since patents are recognized as a quantitative R&D investment performance measure, they are widely used as a technological innovation performance measure [46], and they are useful for technology forecasting [47].

## 3. Methodology

### 3.1 Promising technologies and sub-technologies

Our framework proposing technologies for subcontractors involves three stages. First, we find the technologies with good prospects from the “Management Discussion and Analysis (MD&A)” and “Business” sections of the prime contractors’ business reports. For selecting promising technologies, two conditions need to be satisfied: (1) the technical term that did not appear in the previous year’s business report should appear in the present year’s business report, and (2) it should appear in the present or future positive statements. Then we extract U.S. registered patents through keyword search of these technologies for title, abstract and claims by using WIPSON which is a patent database with a high-quality level in Korea containing full-text information of patents registered in patent offices of 12 countries including big 5 and frequently updating them. The patents found are classified by IPC code, and each group based on IPC code is defined as a sub-technology of the promising technology.

### 3.2 Three factors used for technology selection

Second, from the sub-technologies obtained, we calculate three factors: the market values of the sub-technologies, degree of competition of the sub-technologies in the technology market, and potential technological capabilities of the subcontractors for each sub-technology.

The market values of the sub-technology *i*, *a*_*i*_, are calculated using the patent quality index (PQI), which shows the comprehensive economic and technological value of patents [48]. PQI is strongly linked to the market value of patents [49]. It provides value to the average min-max normalized patent subindex, including forward citations, family size, number of claims, generality index, backward citations, and inverse grant lag. The definition of each patent subindex for PQI is given in Table 1, based on OECD paper [48].

**Table 1:** Description of patent subindex for PQI.

Patent subindex	Description
**Forward citations**	Number of citations a given patent receives (for calculating PQI, we use child patents applied five years after the date of their parent patent publication)
**Family size**	Number of patent offices at which a given invention has been protected
**Number of claims**	Number of claims of the patent, which represents the scope of the patent protection for technology
**Generality index**	$1-\sum_{u}{\left(\frac{\#\;of\;foward\;citations\;in\;the\;classification\;code\;u}{\#\;of\;forward\;citations}\right)}^{2}$ where classification code *u* represents the first four digits of the IPC code, which means the number of technical fields affecting the spread of technology and technology in other areas
**Backward citations**	Number of citations made in a given patent, which can be called a patent reference
**Inverse grant lag**	$1-\frac{grant\;lag}{\max\left\{grant\;lag\;of\;each\;patent\;in\;the\;classification\;code\;u\right\}}$ where classification code *u* represents the first four digits of the IPC code, which means the time taken for a particular patent to be applied for and granted within the technology sector to which the patent belongs (in days)

A patent subindex for PQI is forward citation. It is defined as the number of times a patent has been cited five years after the date of its application. However, if the patent data collected are for the latest period, it is highly likely that the patents considered were applied for less than five years from the present date. Thus, for recent patents, the number of forward citations could be zero. Therefore, we use the zero-inflated negative binomial regression of the patents applied within five years of the present date for predicting the forward citations five years after the date of their application. Zero-inflated negative binomial regression is commonly used for predicting forward citations when their number is zero [50]. This model is found to perform better than a Poisson regression model or negative binomial regression model when zero values are over-distributed and the variance is much greater than the mean [51]. Zero-inflated negative binomial regression is a combination of the count and zero-inflated models, with the former following a negative and the latter following a positive binomial distribution. In this modeling, the number of forward citations is used as dependent variable, and the number of backward citations, number of claims, family size, promising technology to which the patent belongs, and classification code are independent variables as they are commonly used in related studies [52]. We calculate the number of days from the application date to the present point as the offset variable.

The degree of competition of the sub-technology *i* in the technology market, *b*_*i*_, is calculated as the average of the normalized three components, the number of applied patents belonging to the sub-technology, number of companies that applied those patents, and ratio of the number of the sub-technology’s patents to the number of patents with the same classification code.

The potential technological capability of subcontractor *j* for each sub-technology *i*, *c*_*ij*_, is calculated using a cosine similarity between the subcontractor’s patent document abstracts and those of individual sub-technology, summarized using the term frequency-inverse document frequency (TF-IDF) technique. A high similarity shows the possibility of the subcontractors having high technological capability for a particular sub-technology.

TF-IDF considers both how often a word appears and how important each word is in a document [53]. After assigning a weight for each word using the TF-IDF technique, we calculate the cosine similarity between the patent document abstracts of each sub-technology and each technology held by the subcontractors. We select the highest value of the similarities that each patent of each sub-technology has with the patents of a particular subcontractor, and define it as the representative similarity of each patent *m* belonging to a sub-technology *i* for subcontractor *j*, $M_{{\left(i\right)}_{m,},j}$. Then, we define the p-norm value of the representative similarities of all patents belonging to a particular sub-technology *i* as the potential technological capability *c*_*ij*_ that subcontractor *j* has for the sub-technology *i*, as follows:

$$c_{ij}={\left\|M_{ij}\right\|}_{p_{i}}={\left(\sum_{m=1}^{p_{i}}{\left(M_{{\left(i\right)}_{m},j}\right)}^{p_{i}}\right)}^{1/p_{i}}$$ (1)

where *p*_*i*_ is the number of patents belonging to the sub-technology *i* and $M_{ij}=(M_{{\left(i\right)}_{1,},j},M_{{\left(i\right)}_{2},j},\ldots ,M_{{\left(i\right)}_{p_{i}},j})$ is a vector consisting of $M_{{\left(i\right)}_{m},j}$ for all *m* of a particular sub-technology *i* and subcontractor *j*. The p-norm reflects both the average size and maximum value of the components belonging to ***M***_***ij***_. A comparison of the potential technological capability that subcontractor *j* can have for each sub-technology *i* shows that the higher the *c*_*ij*_, the higher is subcontractor *j* judged to have the technological capability potential for the particular sub-technology *i*.

### 3.3 Optimal selection of blue ocean technologies

Finally, using linear programming, we find the optimal solution that maximizes the weighted normalized sum of the three factors for the recommended technology selection. The weight of each factor is determined by the business strategy or circumstances of each subcontractor. The objective function and constraints of individual subcontractor j are as follows:

$$maximize\;\sum_{i}\left[(w_{a}\frac{a_{i}-min(a_{i})}{\max\left(a_{i}\right)-min(a_{i})}+w_{b}\frac{exp({1-b}_{i})-min(exp({1-b}_{i}))}{\max\left(exp({1-b}_{i})\right)-min(exp({1-b}_{i}))}+w_{c}\frac{c_{ij}-min(c_{ij})}{max(c_{ij})-min(c_{ij})})T_{ij}\right]$$ (2)

s.t.

$$T_{ij}=\left\{ \begin{array}{l} 1,\;subtechnology\;i\;is\;selected\;by\;subcontractor\;j\\ 0,subtechnology\;i\;is\;not\;selected\;by\;subcontractor\;j \end{array}\right.$$ (3)

$$\sum_{i}T_{ij}=n$$ (4)

where *w*_*a*_, *w*_*b*_, and *w*_*c*_ are the respective weights of each factor, whose sum is 100, and *n* is the total number of sub-technologies subcontractor *j* plans to choose. As we attempt to find blue ocean technologies, we subtract the competition score *b*_*i*_ from 1 and define it as an inverse competition score. We consider the exponential on 1−*b*_*i*_ because its difference is small.

## 4. Empirical study

In this section, we apply the aforementioned methodology to an actual case of a semiconductor industry. This industry is known as an intellectual property intensive, heavily relying on R&D with rapid technological changes and overall increase in patenting [54,55]. We choose SK Hynix Inc., the world’s third-largest company in terms of revenue among all semiconductor vendors in 2018 [56], as the prime contractor, and some of its subcontractors.

### 4.1 Promising technologies and their sub-technologies

From the business reports for 2018 and 2019 issued by SK Hynix Inc., we found the company’s promising technologies by identifying the technological terms that were not present in the 2018 business report but appeared in the 2019 business report. They are high bandwidth memory, high-end graphics card, foldable display, and high-resolution display. We then extracted the U.S. patents registered from 2017 to 2019 using the keywords of the promising technologies as of June 19, 2020. Thus, we got 245 patents; these have 110 main IPC codes. We classified the patents by their main IPC codes, each representing a sub-technology. The results are presented in Table 2.

**Table 2:** Promising technologies and sub-technologies of SK Hynix Inc.

Promising technology	Patent search keyword	Sub-technology
**High bandwidth memory**	(high) adj1 (bandwidth) adj1 (memory)	G06F-012/00, G06F-003/06, G11C-008/00, G06F-001/10, G06F-001/3287, G06F-012/02, G06F-012/06, G06F-012/0862, G06F-012/0893, G06F-013/00, G06F-013/28, G06F-013/42, G11C-005/02, G11C-005/06, G11C-007/10, G11C-029/00, H01L-023/02, H01L-023/34, H01L-025/16, H01L-025/18, H03K-019/17
**High-end graphics card**	(((highend) or (high-end) or ((high) adj1 (end))) adj1 (graphics or graphic) adj1 (card)) or (((graphics) or (graphic)) adj1 (card))	G06T-001/20, G06F-001/20, G06F-015/80, F28D-001/04, H04L-029/06, B33Y-050/02, G01M-017/00, G06F-001/26, G06F-003/0354, G06F-008/00, G06F-009/455, G06F-009/48, G06F-011/00, G06F-011/22, G06F-012/14, G06F-013/14, G06F-015/16, G06F-015/167, G06K-007/14, G06T-015/00, H04N-007/15, H04N-019/436, H04W-004/00, H05K-001/11, H05K-007/14
**Foldable display**	(foldable) adj1 (display)	G06F-001/16, G06F-003/041, G09G-005/00, H01L-051/00, G02F-001/1333, H05K-005/00, G09F-009/30, H05K-001/00, H05K-001/02, H05K-005/02, G06F-003/042, G06F-003/14, G09G-003/20, H01J-001/62, H05K-007/00, A47F-005/10, C22C-045/00, G09F-001/00, H01L-051/52, B32B-007/12, G05D-023/00, G06F-003/045, G06F-003/0488, G06K-007/10, G09G-003/3208, G09G-003/3225, H01J-001/60, H01L-027/14, H01L-051/05, H01L-051/50, H04M-001/00, H04N-005/232, H04R-003/12, H05B-037/02, H05K-001/18, H05K-005/03, H05K-007/02, H05K-007/10
**High-resolution display**	(high) adj1 (resolution) adj1 (display)	G09G-003/36, H01L-027/32, H04N-007/14, G08B-001/08, G09G-003/00, A61B-003/02, A63F-013/85, G02B-026/02, G02B-027/01, G02F-001/1337, G02F-001/1343, G06F-001/32, G06F-003/041, G06F-003/147, G06T-011/40, G06T-015/00, G09G-005/00, H01L-021/00, H01L-027/12, H01L-029/04, H01L-051/52, H04N-005/232, H04N-005/262, H04N-005/44, H04N-011/02, H04N-019/166

### 4.2 Market values of sub-technologies

**Fig 2:**
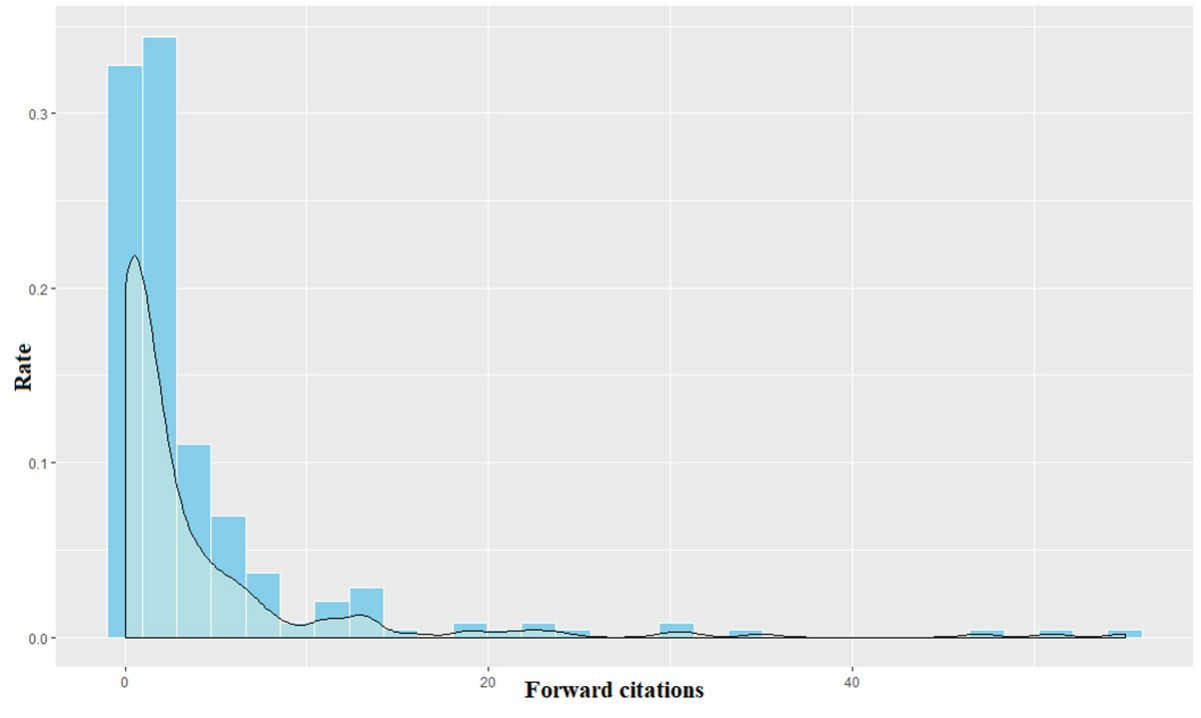
Distribution of forward citations of sub-technologies.

We calculate the PQI values of each sub-technology by the definition given in Table 1. Fig 2 shows the distribution of the observed number of patent forward citations from the date of their application to the date of search in this study. The forward citations of each patent, except for the extreme outlier value 255, have a right skewed distribution of mean 3.816 and variance 57.163.

Because the forward citations of patents showed a right skewed distribution and numerous zero values, we employed zero-inflated negative binomial regression to predict the number of patent forward citations for five years from the patent application date.

A zero-inflated negative-binomial model consists of two sub-models: the count model and zero inflated model. For each model, we used the following independent variables: number of backward citations, number of claims, promising technology, and family size. For the nominal variable of promising technology, high resolution display is set as the baseline category. We used the Akaike Information Criterion (AIC) value for variable selection of each model. Table 3 represents the fitted count model with log link, and Table 4 represents the fitted zero-inflation model with logit link for zero forward citation.

**Table 3:** Fitted count model (negative binomial with log link).

	Estimate	Standard error	Z value	P value
**Intercept**	-7.958	0.178	-44.636	<0.001
**High bandwidth memory**	-0.419	0.248	-1.689	0.091
**High -end graphics card**	-0.397	0.213	-1.865	0.062
**Foldable display**	1.363	0.109	12.547	<0.001
**family size**	0.053	0.015	3.558	<0.001
**Backward Citation**	0.006	0.0006	8.963	<0.001
**Claim**	0.064	0.005	12.125	<0.001

**Table 4:** Fitted zero-inflation model (binomial with logit link).

	Estimate	Standard error	Z value	P value
**Intercept**	-8.787	0.241	-36.516	<0.001
**Backward Citation**	0.005	0.003	1.955	0.051

Table 5 presents the descriptive statistics of the sub-technology PQI values classified by promising technologies. We find that the display sectors generally have larger PQI values than the rest. This indicates that more sub-technologies generally have higher market values in display sectors than in other sectors on average.

**Table 5:** Descriptive statistics of PQI.

Promising technology	PQI			
	Mean	Max	Min	Variance
**High bandwidth memory**	0.276	0.486	0.125	0.006
**High-end graphics card**	0.254	0.341	0.175	0.002
**Foldable display**	0.404	0.703	0.282	0.006
**High resolution display**	0.351	0.522	0.171	0.008

### 4.3 Degree of competition between sub-technologies in the technology market

We calculate the competition score of each sub-technology. To summarize the results, Table 6 shows the descriptive statistics of the sub-technologies’ competition scores classified by promising technologies.

**Table 6:** Descriptive statistics of competition score.

Promising technology	Competition score			
	Mean	Max	Min	Variance
**High bandwidth memory**	0.032	0.167	0.001	0.002
**High-end graphics card**	0.028	0.133	0.000	0.001
**Foldable display**	0.057	0.673	0.000	0.013
**High resolution display**	0.033	0.333	0.000	0.005

### 4.4 Potential technological capabilities of subcontractors for each sub-technology

We chose three subcontractors of SK Hynix Inc.—Daeduck Electronics, Soulbrain, and UniTest. They are selected as they are financially stable and superior subcontractors that are suitable for challenging new technology adoption or expansion. Daeduck Electronics is an electric and electronics company mainly dealing with the manufacture of laminated printed circuit board plates. Soulbrain is a semiconductor and display company mainly engaged in chemical manufacturing. UniTest is a machinery and equipment company mainly manufacturing machines for semiconductors. These companies focus on domestic business, and their patents were disclosed or granted by the Korean Intellectual Property Office (KIPO) from 2017 to 2019. The p-norm of maximum similarity between the patents of each sub-technology and subcontractor indicates the potential technological capability of each subcontractor for each sub-technology.

**Fig 3:**
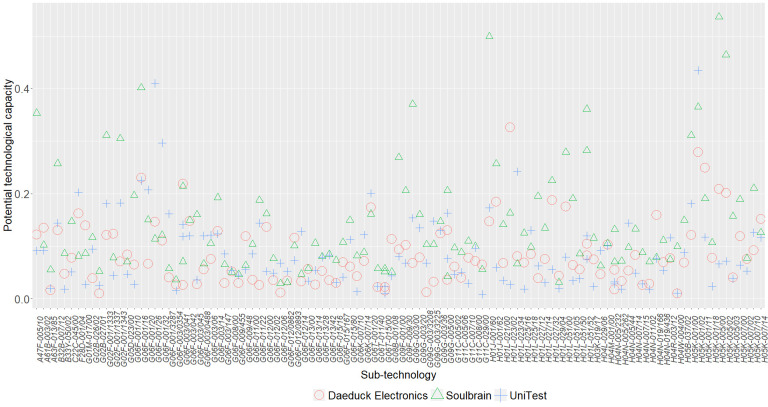
Potential technological capability of each subcontractor for sub-technologies.

Table 7 lists the descriptive statistics of the potential technological capabilities classified by promising technology. Fig 3 displays the potential technological capability of each subcontractor for each sub-technology. Daeduck Electronics shows the highest technological capability in the high bandwidth memory sector, whereas both Soulbrain and UniTest show the highest technological capability in the foldable display sector. Daeduck Electronics has the highest technological capability in H01L-023/02 of the high bandwidth memory sector, Soulbrain in H05K-005/00 of the foldable display sector, and UniTest in H05K-001/02 of the foldable display sector, with 0.326, 0.537, and 0.435, respectively.

**Table 7:** Descriptive statistics of potential technological capability.

	Potential technological capability											
	Daeduck Electronics				Soulbrain				UniTest			
	mean	max	min	std	mean	max	min	std	Mean	max	Min	std
**High bandwidth memory**	0.077	0.326	0.011	0.064	0.095	0.197	0.030	0.042	0.070	0.242	0.009	0.056
**High-end graphics card**	0.075	0.249	0.010	0.062	0.099	0.191	0.036	0.043	0.095	0.410	0.010	0.089
**Foldable display**	0.109	0.279	0.012	0.062	0.212	0.537	0.067	0.122	0.101	0.435	0.014	0.075
**High resolution display**	0.068	0.187	0.010	0.049	0.122	0.371	0.032	0.095	0.078	0.296	0.018	0.065

**Fig 4:**
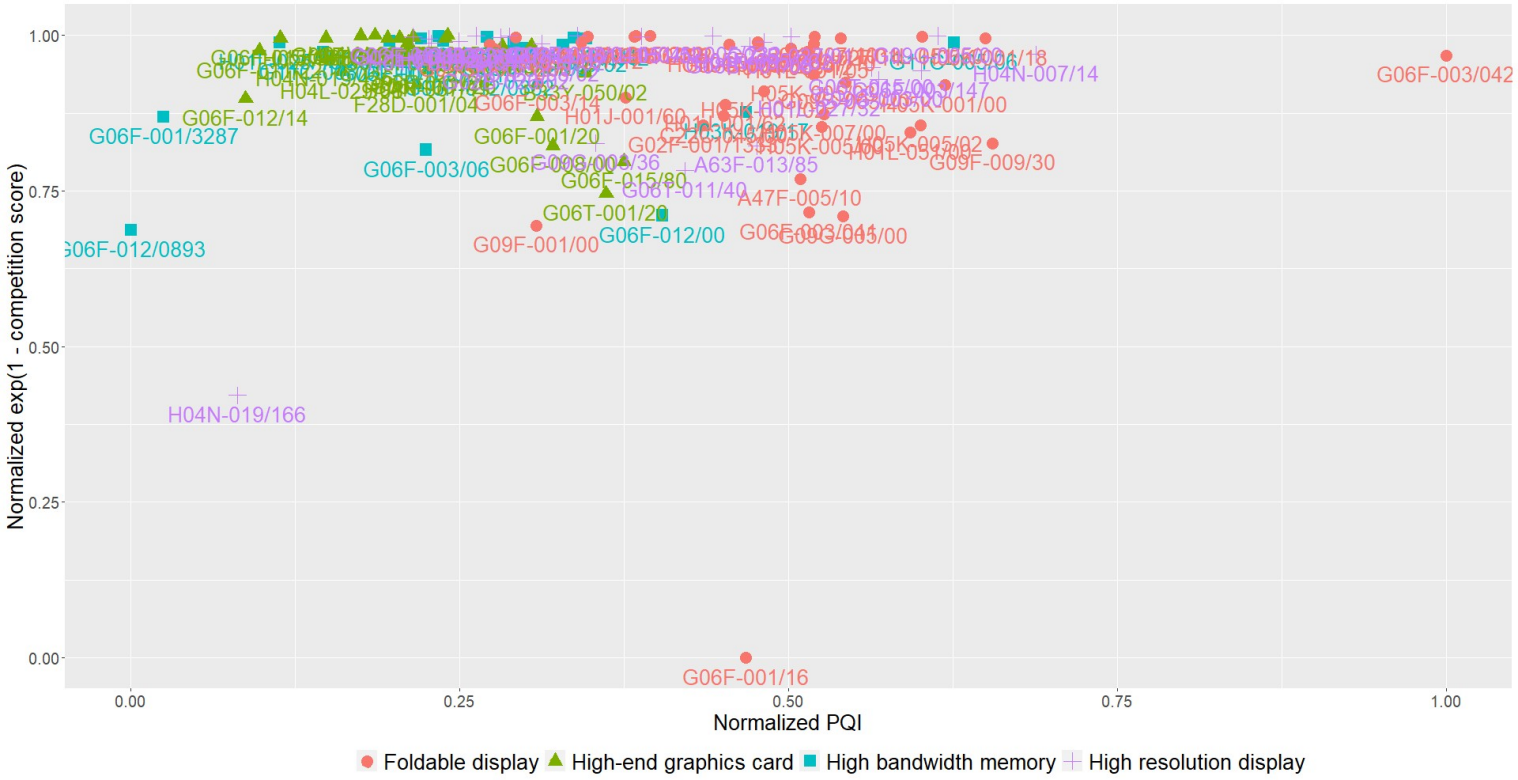
Mapping of the market value and competition of sub-technologies.

To evaluate the sub-technologies themselves as blue ocean technologies, Fig 4 shows a sub-technology map of normalized values for PQI and exponential value of one minus competition score as coordinates.

Most of the sub-technologies, especially those in the high-end graphics card sector, are clustered near a coordinate of (0.25, 1.00). However, the sub-technologies in the foldable display sector are also widespread in areas where the normalized PQI is relatively high.

### 4.5 Optimal selection of blue ocean technologies

We carry out a sensitivity analysis on how the recommended sub-technologies change by controlling the objective functional weight in linear programming. We find the optimal solution by setting each weight to at least 20% of the total weight and changing the weight in 10% units of the total weight. Table 8 shows the recommended sub-technologies as optimal solutions following the change in weight, which resulted in change in the recommended sub-technologies for subcontractors. Therefore, we find that the decisions on technology development and expansion can vary depending on the strategies the subcontractors adopt. If the subcontractor pursues mainstream technologies rather than self-potential capability, it would provide a higher weight to the market value and inverse competition scores. In contrast, if the subcontractor focuses more on its main technology, it would grant its potential technological capability a higher weight.

**Table 8:** Recommended sub-technologies by controlling weights.

Weight			Recommended sub-technologies								
*w*_*a*_	*w*_*b*_	*w*_*c*_	Daeduck Electronics			Soulbrain			UniTest		
			Rank 1	Rank 2	Rank 3	Rank 1	Rank 2	Rank 3	Rank 1	Rank 2	Rank 3
**2**	**2**	**6**	H01L-023/02	H05K-001/02	H05K-001/11	H05K-005/00	H01J-001/60	H05K-005/02	H05K-001/02	G06F-001/26	G06F-001/32
**2**	**3**	**5**	H01L-023/02	H05K-001/02	G06F-003/042	H05K-005/00	H01J-001/60	H05K-005/02	H05K-001/02	G06F-001/26	G06F-001/32
**2**	**4**	**4**	H01L-023/02	H05K-001/02	G06F-003/042	H05K-005/00	H01J-001/60	H05K-005/02	H05K-001/02	G06F-001/26	G06F-001/32
**2**	**5**	**3**	H01L-023/02	G06F-003/042	H05K-001/02	H05K-005/00	H05K-005/02	H01J-001/60	H05K-001/02	G06F-001/26	G06F-003/042
**2**	**6**	**2**	G06F-003/042	H01L-023/02	H05K-001/02	G06F-003/042	H05K-005/00	H01L-051/52	H05K-001/02	G06F-003/042	G06F-001/26
**3**	**2**	**5**	H01L-023/02	H05K-001/02	G06F-003/042	H05K-005/00	H05K-005/02	H01J-001/60	H05K-001/02	G06F-001/26	G06F-003/042
**3**	**3**	**4**	G06F-003/042	H01L-023/02	H01L-023/02	H05K-005/00	H05K-005/02	H01J-001/60	H05K-001/02	G06F-001/26	G06F-003/042
**3**	**4**	**3**	G06F-003/042	H05K-001/02	H01L-023/02	H05K-005/00	H05K-005/02	G06F-003/042	H05K-001/02	G06F-003/042	G06F-001/26
**3**	**5**	**2**	G06F-003/042	H05K-001/02	H01L-023/02	G06F-003/042	H05K-005/00	H05K-005/02	G06F-003/042	H05K-001/02	G06F-001/26
**4**	**2**	**4**	G06F-003/042	H05K-001/02	H01L-023/02	H05K-005/00	H05K-005/02	H01J-001/60	H05K-001/02	G06F-003/042	G06F-001/26
**4**	**3**	**3**	G06F-003/042	H05K-001/02	H05K-005/02	H05K-005/00	G06F-003/042	H05K-005/02	G06F-003/042	H05K-001/02	G06F-001/26
**4**	**4**	**2**	G06F-003/042	H05K-001/02	H04N-007/14	G06F-003/042	H05K-005/02	H05K-005/00	G06F-003/042	H05K-001/02	H05K-001/00
**5**	**2**	**3**	G06F-003/042	H05K-001/02	H04N-007/14	G06F-003/042	H05K-005/02	H05K-005/00	G06F-003/042	H05K-001/02	H05K-001/00
**5**	**3**	**2**	G06F-003/042	H05K-001/02	H04N-007/14	G06F-003/042	H05K-005/02	H05K-005/00	G06F-003/042	H05K-001/02	H05K-001/00
**6**	**2**	**2**	G06F-003/042	H04N-007/14	H05K-005/02	G06F-003/042	H05K-005/02	H05K-005/00	G06F-003/042	H05K-001/02	H05K-001/00

The technology recommended for all the three subcontractors is G06F-003/042 (digitizers such as those for touch screens or touch pads, characterized by opto-electronic means), which has the highest PQI and is therefore frequently recommended as top-tier technology, especially when we give high weights to the market value or competition score. This means that we can consider the technology itself as a blue ocean, regardless of the potential technological capability of the subcontractors. In fact, digitizer-related technology shows higher marketability in its similar technology group, as the average number of patent family countries with IPC code starting with G06F registered in the first half of 2020 is 2.63, compared to 3.98 for patents with IPC code G06F-003/042.

For Daeduck Electronics, when the lowest weight, 20%, is given to the market value of technologies, H01L-023/02 (semiconductor containers or seals or other solid-state devices over which H01L 23/12, H01L 23/34, H01L 23/48, and H01L 23/552 take precedence) is mostly ranked the highest. This technology tends to show an improvement in ranking as the weight of the market value decreases. When the highest weight, 60%, is given to technological capability, H05K-001/11 (printed elements for providing electric connections to or between printed circuits) is the newly detected technology recommended. Regardless of weight, H05K-001/02 (details of printed circuits) is mostly in the list of the top three recommended technologies.

For Soulbrain, when the weight of the market value is decreased, H05K-005/00 (casings, cabinets, or drawers for electric apparatus) and H01J-001/60 (incandescent screens on or from which an image or pattern is formed, picked up, converted, or stored) are prominently detected in the top three rankings, indicating an improvement in rankings. H05K-005/00 and H05K-005/02 (details of casings, cabinets, or drawers for electric apparatus) are always in the top three ranks regardless of weight.

For UniTest, when the weight of the market value of technologies is not the highest, G06F-001/26 (power supply means, such as regulation thereof for memories G11C) is prominently detected in the top three rankings. In particular, when the lowest weight, 20%, is given to the market value of technologies, G06F-001/32 (power supply means for saving power) is the newly detected recommended technology in the top three ranks. H05K-001/02 (details of printed circuits) is always in the top three ranks regardless of weight.

## 5. Discussion

Through the empirical study, we identified recommended technologies for individual subcontractors with changes in the weights of three factors. It backs up the studies that decisions on the expansion or development of technologies can vary depending on the subcontractors’ strategy and direction [57,58]. There are two cases: (1) a commonly recommended technology for all the subcontractors and (2) technologies recommended differently for each subcontractor. The former supports previous studies that it is important to track and identify the technology trend for business survival [59,60]. It can explain why different subcontractors can be recommended with the same technology in common. The latter supports existing studies that a new technology needs to be chosen not only by the value of the technology itself, but also by the subcontractor’s current technology level and potential technological capability [1,21,26,41].

This study provides insights to the existing studies [15,16,18], and the existing problem in subcontracting where win-win relationship between subcontractors and their prime contractors has not been considered carefully. Previous studies have focused on subcontractors being chosen by prime contractors while have not fully considered subcontractors’ technological followership to their prime contractors. The contribution of our paper is to consider their win-win relationship with reflecting the subcontractors’ technological followership to their prime contractors, which makes the risk of subcontractors’ technology development lower. This is why our study contributed to the existing problem in subcontracting.

## 6. Conclusions

This study proposed a framework for subcontractors so as to choose blue ocean technologies based on a win-win relationship with their prime contractors. In this framework, one finds promising technologies from the business reports of the prime contractor and classifies the IPC codes of patents searched by keywords of the promising technologies to identify sub-technologies. Then three factors are used to optimize the sub-technology-selection process: market values of the sub-technologies, degree of competition of the sub-technologies in the technology market, and potential technological capabilities of the subcontractors for each sub-technology. These factors are measured by the PQI; average of the normalized number of applied patents of the sub-technology, number of companies that applied those patents, and ratio of number of sub-technology patents to number of patents with the same classification code; and the cosine similarity with the TF-IDF technique, respectively. The proposed framework is applied to the case of the subcontractors of SK Hynix Inc.: Daeduck Electronics, Soulbrain, and UniTest.

As a result, we found out following. Digitizer-related technology such as G06F-003/042 was commonly recommended to those subcontractors, as it has a high score as a blue ocean technology. On the other hand, different technologies were recommended depending on the potential technological capability of each subcontractor by increasing the weight of the technological capability factor. Technologies related to containers or seals of semiconductor (H01L-023/02), incandescent screens (H01J-001/60), power supply means (G06F-001/26) were respectively suggested to Daeduck Electronics, Soulbrain, and UniTest as a differentiation strategy.

This study used a combination of qualitative and quantitative approaches to help the subcontractors decide on developing new technologies and expanding their existing technologies, which benefit both subcontractors and their prime contractors. Due to the imbalance of power between prime and subcontractors, subcontractors have not had initiative to innovate themselves but had to rely on the prime contractors’ needs. However, we suggest the three criteria (market value of technology, degree of competition in the market, and the suitability of subcontractors to develop promising technologies) for subcontractors to develop blue ocean technologies that can benefit both the prime and subcontractors. Subcontractors may adjust the weight of three factors based on common interest of them and their prime contractors. Externally, our framework fully reflects the subcontractors’ business relationship with their prime contractors and reduces their risks of technology development. Internally, the framework recommends technologies considering the subcontractors’ technological capabilities. Therefore, by applying the methodology of this study, subcontractors can have a positive impact on their own as well as their prime contractors’ innovation by developing and expanding the recommended technologies. This study thus enables subcontractors to have a long-term technological competitive edge and coexist with their prime contractor as well as positively contribute as technology providers to the supply chain.

However, the study has following limitations. In the empirical study, there was an issue of small sample size, as some promising technologies do not have many patents. In predicting forward citations, one of subindices for PQI, we could not include more diverse independent variables. Extension to these areas is left for further research.

## Supporting information

S1 Dataset Patent dataset for this paper. (XLSX)

## References

[1] Arditi D, Chotibhongs R. Issues in subcontracting practice. *Journal of Construction Engineering and Management*, 2005, 131.8: 866–876.

[2] Eggert A, Böhm E, Cramer C. Business service outsourcing in manufacturing firms: An event study. *Journal of Service Management*, 2017, 28.3: 476–498.

[3] Seppänen V. Evolution of competence in software subcontracting projects. *International Journal of Project Management*, 2002, 20.2: 155–164.

[4] Chen Q, Jin R. A comparison of subgroup construction workers’ perceptions of a safety program. *Safety Science*, 2015, 74: 15–26.

[5] Gilley KM, Rasheed A. Making more by doing less: an analysis of outsourcing and its effects on firm performance. *Journal of Management*, 2000, 26.4: 763–790.

[6] Jeon J, Hong S, Ohm J, Yang T. Causal relationships among technology acquisition, absorptive capacity, and innovation performance: evidence from the pharmaceutical industry. *PloS One*, 2015, 10.7: e0131642. doi: 10.1371/journal.pone.0131642 26181440 PMC4504511

[7] Qi X. Production scheduling with subcontracting: the subcontractor’s pricing game. *Journal of Scheduling*, 2012, 15.6: 773–781.

[8] Crespi G, Katz J, Olivari J. Innovation, natural resource-based activities and growth in emerging economies: the formation and role of knowledge-intensive service firms. *Innovation and Development*, 2018, 8.1: 79–101.

[9] Sousa R, Voss CA. Operational implications of manufacturing outsourcing for subcontractor plants. *International Journal of Operations & Production Management*, 2007.

[10] Andersen PH. Organizing international technological collaboration in subcontractor relationships: an investigation of the knowledge-stickiness problem. *Research Policy*, 1999, 28.6: 625–642.

[11] Laursen K, Salter A. Open for innovation: the role of openness in explaining innovation performance among UK manufacturing firms. *Strategic Management Journal*, 2006, 27.2: 131–150.

[12] Dougherty D, Hardy C. Sustained product innovation in large, mature organizations: Overcoming innovation-to-organization problems. *Academy of Management Journal*, 1996, 39.5: 1120–1153.

[13] Nwokocha VC, Nwankwo C, Madu IA. The role of subcontracting on innovation: an assessment of small and medium enterprises in Nigeria. *Production & Manufacturing Research*, 2019, 7.1: 88–108.

[14] Martínez-Noya A, García-Canal E. Location, shared suppliers and the innovation performance of R&D outsourcing agreements. *Industry and Innovation*, 2018, 25.3: 308–332.

[15] McCord PJ. *Subcontractor Perspectives: Factors that Most Affect Their Relationships with General Contractors, a Pacific Northwest Study*. 2010. PhD Thesis. Washington State University.

[16] Sun Y, Zhou Y, Lin GC, Wei YD. Subcontracting and supplier innovativeness in a developing economy: evidence from China’s information and communication technology industry. *Regional Studies*, 2013, 47.10: 1766–1784.

[17] Gold S, Chesney T, Grunchmann T, Trautrisms A. Diffusion of labor standards through supplier–subcontractor networks: An agent‐based model. *Journal of Industrial Ecology*, 2020, 24.6: 1274–1286.

[18] Yun M. Subcontracting relations in the Korean automotive industry: risk sharing and technological capability. *International Journal of Industrial Organization*, 1999, 17.1: 81–108.

[19] Miller CJM, Packham GA, Thomas BC. Harmonization between main contractors and subcontractors: a prerequisite for lean construction?. *Journal of Construction Research*, 2002, 3.01: 67–82.

[20] Contasfor Egan SJ, Willams D. Rethinking Construction-The Report of the Construction Task Force. Ice Briefing Sheet. In: *Proceedings of the Institution of Civil Engineers-Municipal Engineer*. Thomas Telford-ICE Virtual Library, 1998. p. 199–203.

[21] Pardo R, RAMA R. Is the Pro-Network Bias Justified? Outsourcers, Small Batch Production, and Advanced Manufacturing Technology. *SAGE Open*, 2013, 3.3: 2158244013497032.

[22] Kim HJ, Kim TS, Sohn SY. Recommendation of startups as technology cooperation candidates from the perspectives of similarity and potential: A deep learning approach. *Decision Support Systems*, 2020, 130: 113229.

[23] Adner R, Kapoor R. Value creation in innovation ecosystems: How the structure of technological interdependence affects firm performance in new technology generations. *Strategic Management Journal*, 2010, 31.3: 306–333.

[24] Cao D, Wang G. Contractor–subcontractor relationships with the implementation of emerging interorganizational technologies: Roles of cross-project learning and pre-contractual opportunism. *International Journal of Construction Education and Research*, 2014, 10.4: 268–284.

[25] Hafeez S, Arshad NI, Rahim LBA, Shabbir MF, Iqbal J. Innovation in Chinese internet companies: A meta-frontier analysis. *Plos One*, 2020, 15.5: e0233278. doi: 10.1371/journal.pone.0233278 32437383 PMC7241703

[26] Mukherji N, Rajagopalan B, Tanniru M. A decision support model for optimal timing of investments in information technology upgrades. *Decision Support Systems*, 2006, 42.3: 1684–1696.

[27] Zhu K, Weyant JP. Strategic decisions of new technology adoption under asymmetric information: a game‐theoretic model. *Decision Sciences*, 2003, 34.4: 643–675.

[28] Rosenbusch N, Brinckmann J, Bausch A. Is innovation always beneficial? A meta-analysis of the relationship between innovation and performance in SMEs. *Journal of Business Venturing*, 2011, 26.4: 441–457.

[29] Tong Zhao, Song Z, Li T. Effect of innovation capacity, production capacity and vertical specialization on innovation performance in China’s electronic manufacturing: Analysis from the supply and demand sides. *PloS One*, 2018, 13.7: e0200642. doi: 10.1371/journal.pone.0200642 30011311 PMC6065606

[30] Lerner J. The future of public efforts to boost entrepreneurship and venture capital. *Small Business Economics*, 2010, 35.3: 255–264.

[31] Laryea S, Lubbock A. Tender pricing environment of subcontractors in the United Kingdom. *Journal of Construction Engineering and Management*, 2014, 140.1: 04013029.

[32] Veloso F, Fixson S. Make–buy decisions in the auto industry: new perspectives on the role of the supplier as an innovator. *Technological Forecasting and Social Change*, 2001, 67.2–3: 239–257.

[33] Ko CH. Predicting subcontractor performance using web-based evolutionary fuzzy neural networks. *The Scientific World Journal*, 2013, 2013. doi: 10.1155/2013/729525 23864830 PMC3705785

[34] Goel RK, Rehman F. What induces firms to subcontract to the informal sector? Evidence from a developing country. *Applied Economics Letters*, 2020, 27.3: 178–187.

[35] Leitch RM. The causes of prime contractor effort: lessons learned from spacecraft procurements. In: *Proceedings*. *2005 IEEE International Engineering Management Conference, 2005*. IEEE, 2005. p. 887–891.

[36] Skowronski K, Benton WC Jr. The influence of intellectual property rights on poaching in manufacturing outsourcing. *Production and Operations Management*, 2018, 27.3: 531–552.

[37] Mazzola E, Bruccoleri M, Perrone G. The curvilinear effect of manufacturing outsourcing and captive-offshoring on firms’ innovation: the role of temporal endurance. *International Journal of Production Economics*, 2019, 211: 197–210.

[38] Furlan A, Grandinetti R, Camuffo A. How do subcontractors evolve?. *International Journal of Operations & Production Management*, 2007.

[39] Sturgeon TJ. Modular production networks: a new American model of industrial organization. *Industrial and Corporate Change*, 2002, 11.3: 451–496.

[40] Singleton T, Cormican K. The influence of technology on the development of partnership relationships in the Irish construction industry. *International Journal of Computer Integrated Manufacturing*, 2013, 26.1–2: 19–28.

[41] Radway R, Helmersson A, Melander A. Facing the global economic crisis: the case of Swedish heavy vehicle subcontractors. *International Journal of Automotive Technology and Management*, 2011, 11.3: 269–293.

[42] Kim WC, Mauborgne R. Value innovation: a leap into the blue ocean. *Journal of Business Strategy*, 2005.

[43] Kim WC, Mauborgne R. Blue Ocean Strategy. *Harvard Business Review*. Retrieved August, 2004, 1: 2014. 15559577

[44] Huang L, Xu Y. A approach to identify the commercialization potential of new technology. In: *2010 International Conference on Education and Management Technology*. IEEE, 2010. p. 329–333.

[45] Nitin Aggarwal, Walden EA. Intellectual Property Bundle (IPB) theory: Managing transaction costs in technology development through network governance. *Decision Support Systems*, 2009, 48.1: 23–32.

[46] Lee HH, Zhou J, Hsu PH. The role of innovation in inventory turnover performance. *Decision Support Systems*, 2015, 76: 35–44.

[47] Sohn SY, Ahn BJ. Multigeneration diffusion model for economic assessment of new technology. *Technological Forecasting and Social Change*, 2003, 70.3: 251–264.

[48] Squicciarini M, Dernis H, Criscuolo C. Measuring patent quality: Indicators of technological and economic value. *OECD Science*, *Technology and Industry Working Papers*, 2013. Available from: 10.1787/5k4522wkw1r8-en.

[49] Lanjouw JO, Schankerman M. Patent quality and research productivity: Measuring innovation with multiple indicators. *The Economic Journal*, 2004, 114.495: 441–465.

[50] Chen C. Predictive effects of structural variation on citation counts. *Journal of the American Society for Information Science and Technology*, 2012, 63.3: 431–449.

[51] Choi JH, Ko IM, Cheon SY. A Zero-Inated Model for Insurance Data. *The Korean Journal of Applied Statistics*, 2011, 24.3: 485–494.

[52] Lee YG, Lee JD, Song YI, Lee SJ. An in-depth empirical analysis of patent citation counts using zero-inflated count data model: The case of KIST. *Scientometrics*, 2007, 70.1: 27–39.

[53] Ramos J. Using tf-idf to determine word relevance in document queries. In: *Proceedings of the first Instructional Conference on Machine Learning*. 2003, p. 29–48.

[54] Alcácer J, Zhao M. Local R&D strategies and multilocation firms: The role of internal linkages. *Management Science*, 2012, 58.4: 734–753.

[55] Forti E, Morricone S, Munari F. Litigation risks and firms innovation dynamics after the IPO. *Journal of Industrial and Business Economics*, 2021, 48.2: 291–313.

[56] Jennifer G, Gloria O. Gartner Says Worldwide Semiconductor Revenue Grew 13.4 Percent in 2018; Increase Driven by Memory Market. *Gartner Newsroom*. 2019 Jan 19 [Cited 2020 July 6]. Available from: https://www.gartner.com/account/signin?method=initialize&TARGET=http%253A%252F%252Fwww.gartner.com%252Fdocument%252F3895910.

[57] Çetindamar D, Phaal R, Probert D. *Technology management*: *activities and tools*. Macmillan International Higher Education. 2016 Jan 20.

[58] Burgelman RA, Maidique MA, Wheelwright SC. *Strategic management of technology and innovation*. Vol 2. Chicago: Irwin, 1996. doi: 10.1159/000170297 8915573

[59] Dereli T, Durmuşoğlu A. Classifying technology patents to identify trends: applying a fuzzy-based clustering approach in the Turkish textile industry. *Technology in Society*, 2009, 31.3: 263–272.

[60] Noh H, Lee S. What constitutes a promising technology in the era of open innovation? An investigation of patent potential from multiple perspectives. *Technological Forecasting and Social Change*, 2020, 157: 120046.

